# Supplementary material for: pH-Dependent Structural Dynamics of Cathepsin D-Family Aspartic Peptidase of Clonorchis sinensis
Source: Pathogens. 2021 Sep 2;10(9):1128. doi: 10.3390/pathogens10091128 (PMC8466142; doi:10.3390/pathogens10091128)
Supplement: Supplementary file 1 [file pathogens-10-01128-s001.zip › Supplementary_materials_v2.pdf]

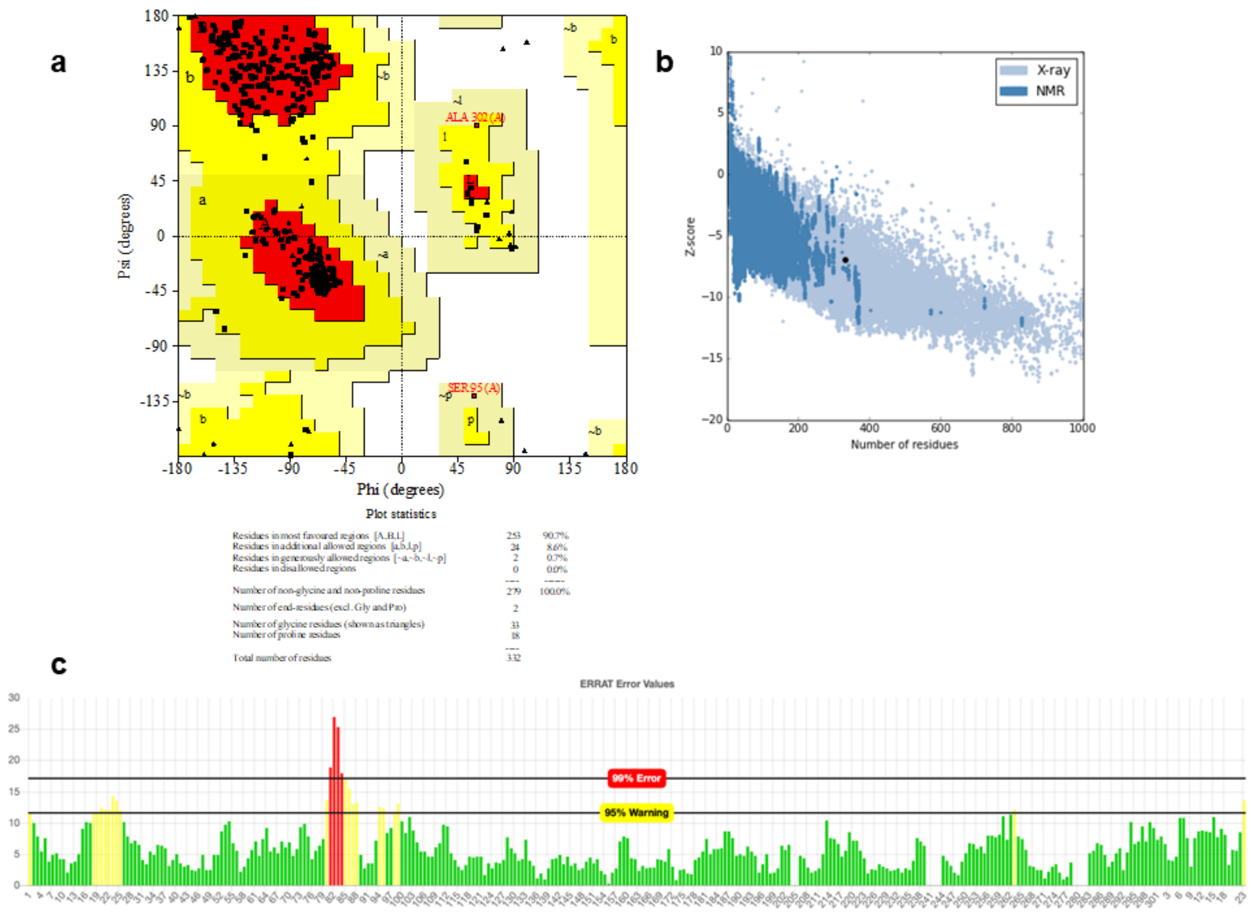


**Figure S1.** Quality verification of tertiary model of mature CsCatD2$_{free}$. (**a**) Ramachandran plot [1] showing the residues in the most favored regions (90.7%), additional allowed regions (8.6%), generously allowed regions (0.7%), and disallowed regions (0%). Red (A, B, L), yellow (a, b, l, p) and light yellow (~a, ~b, ~l, ~p) indicate the most favored regions, allowed regions and generously allowed regions. White shows disallowed regions. All non-glycine and non-proline residues are shown as closed black squares while glycines (non-end) are shown as closed black triangles. Disallowed residues are colored in red. (**b**) The ProSA energy profile [2] indicates that the Z-score was −6.95. (**c**) In the ERRAT plot [3], the overall quality factor is 92.36%.

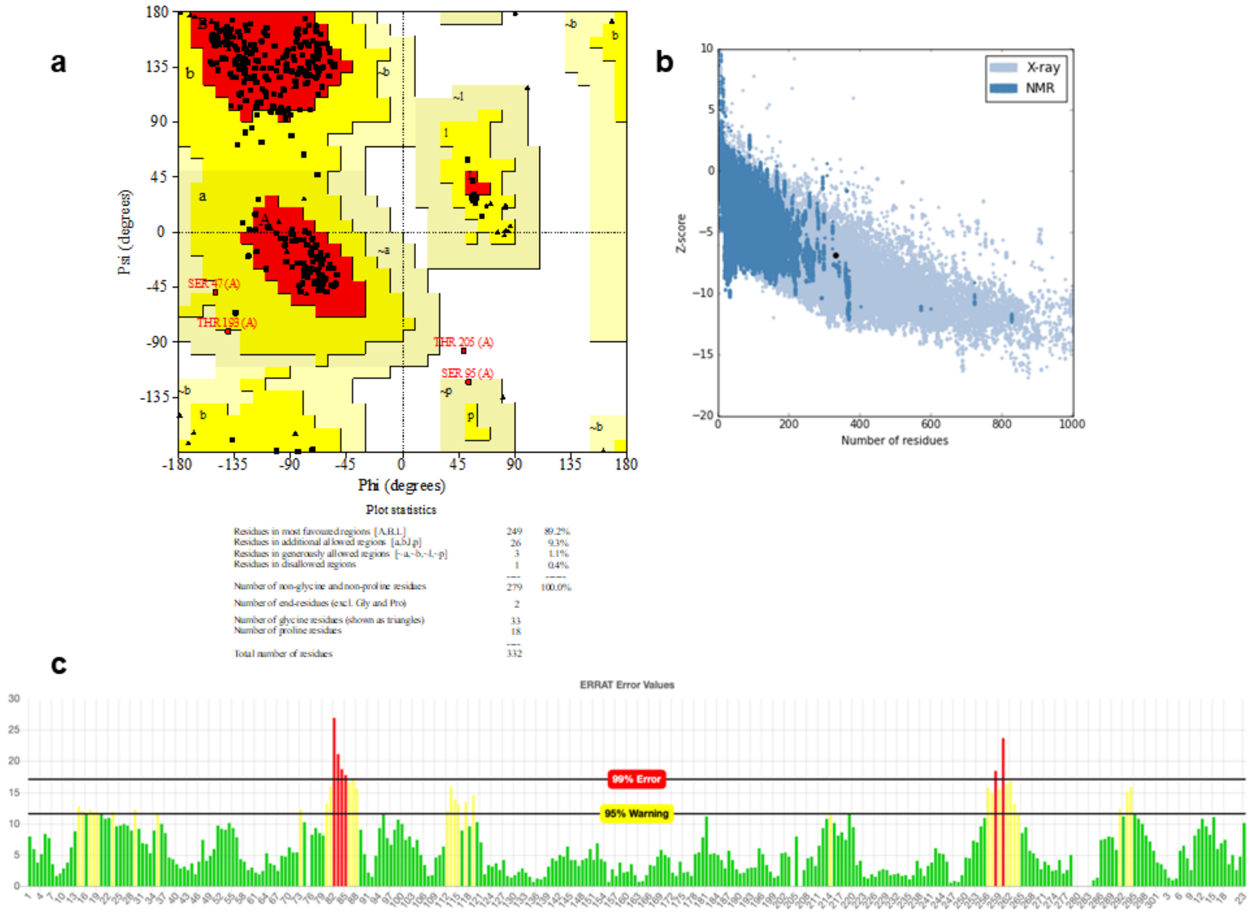


**Figure S2.** Quality verification of tertiary model of CsCatD2bound. (**a**) Ramachandran plot [1] showing the residues in the most favored regions (89.2%), additional allowed regions (9.3%), generously allowed regions (1.1%), and disallowed regions (0.4%). Description regarding the resultant figure is the same as shown in Figure S1. (**b**) The ProSA energy profile [2] indicates that the Z-score was −6.86. (**c**) In the ERRAT plot [3], the overall quality factor is 88.29%.

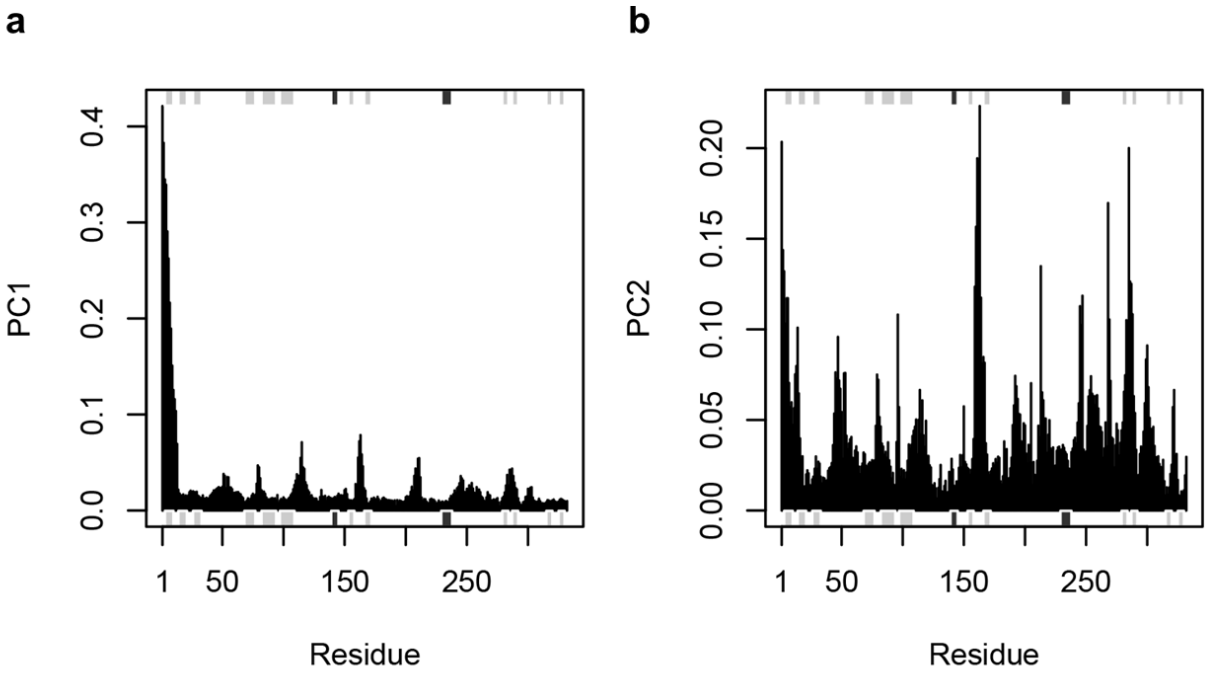


**Figure S3.** Contribution of each residue of CsCatD2 to the PC1 (**a**) and PC2 (**b**). Principal components analysis (PCA) analysis of 28 snapshots during 30–36 ns MD simulation. Secondary structure elements were predicted using DSSP (https://swift.cmbi.umcn.nl/gv/dssp/). Colors and alphabets identify $\alpha$-helices in black and β-strands in gray.

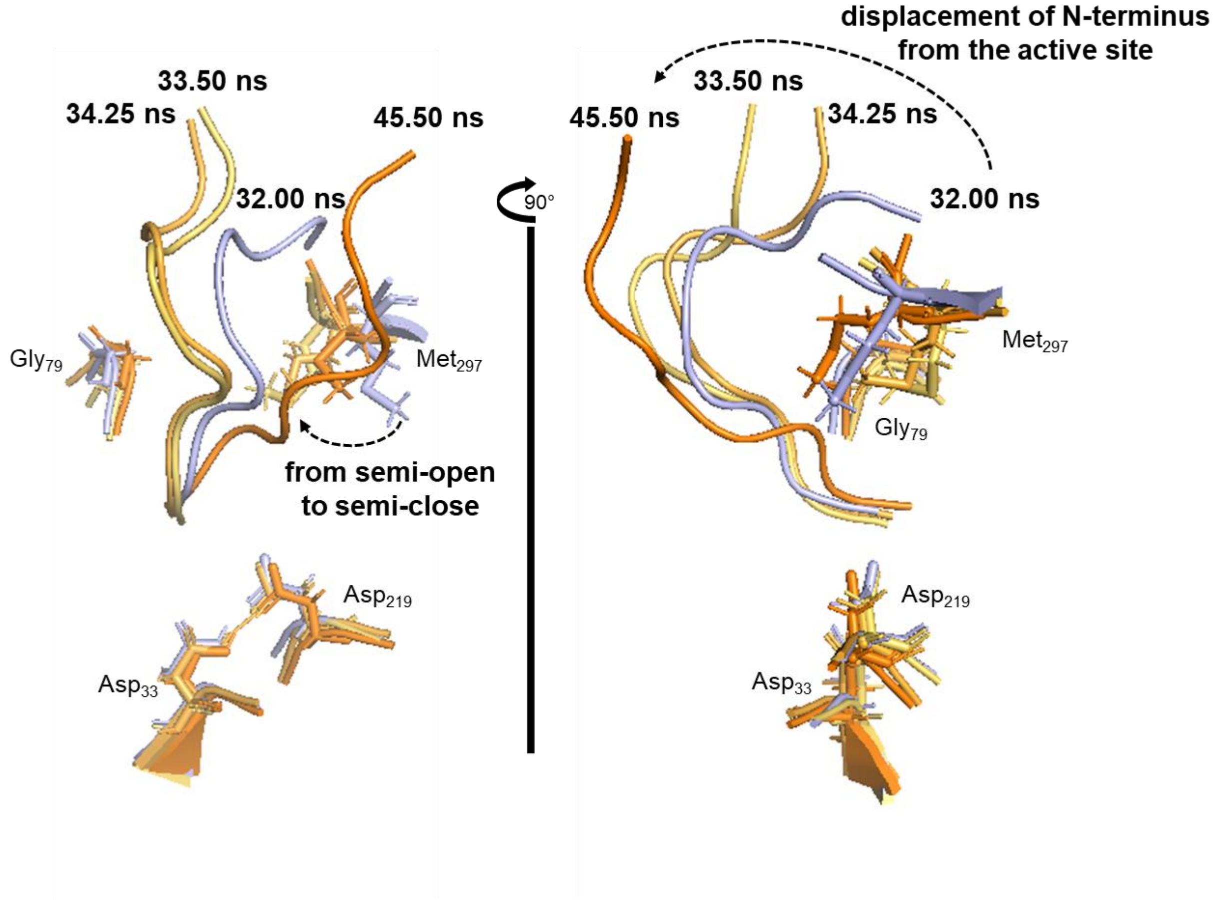


**Figure S4**. Snapshots of CsCatD2$_{free}$ at pH 4 at different time-points 32.0, 33.50, 34.25 and 45.50 ns. (See Section 2.8 for more information about open state.)

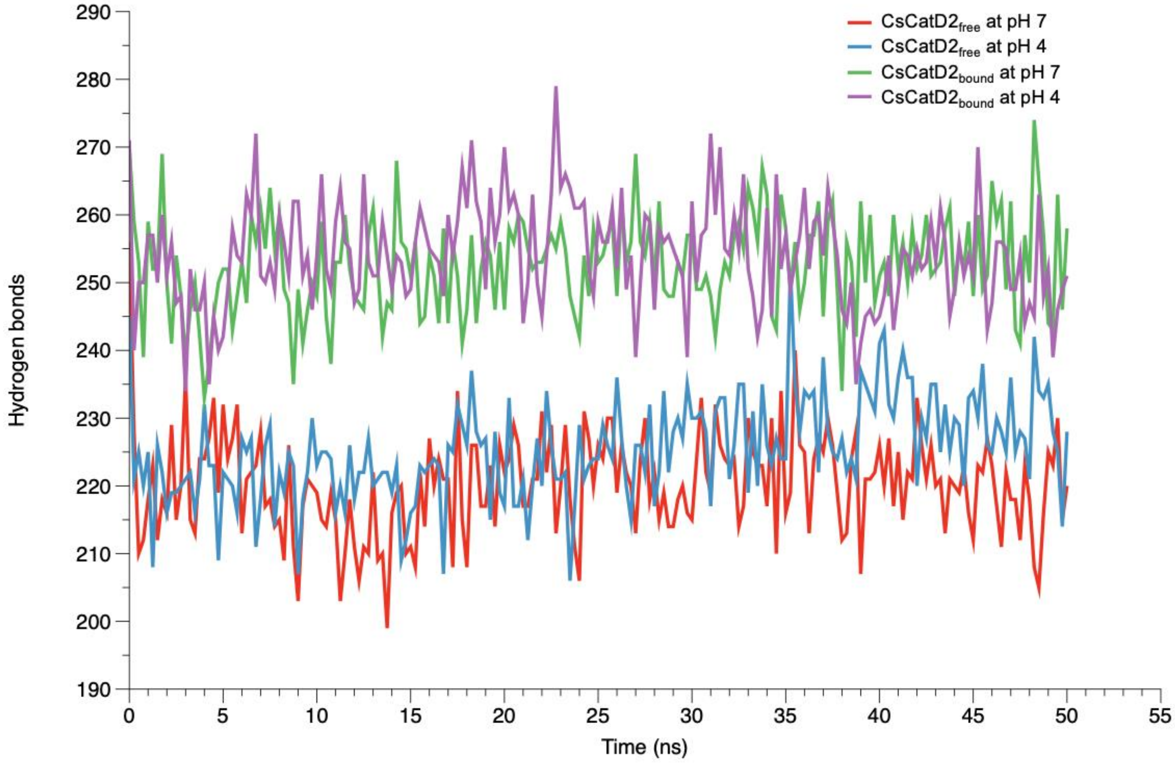


**Figure S5**. Variations of the number of hydrogen bonds through the course of 50-ns MD simulation.

## TM-align results

```
Name of Chain_1: free CsCatD2
Name of Chain_2: free IrCatD (PDB code: 5n7n)
Length of Chain_1: 332 residues
Length of Chain_2: 361 residues

Aligned length=  329, RMSD=  1.76, Seq_ID=n_identical/n_aligned= 0.505
TM-score= 0.93281 (if normalized by length of Chain_1)
TM-score= 0.86117 (if normalized by length of Chain_2)
(You should use TM-score normalized by length of the reference protein)

(":" denotes aligned residue pairs of d < 5.0 A, "." denotes other aligned residues)
                                      ***               *
------------------------QPFPEK-LDNYMDSQYYGEIAIGTPPQPFKVVFDTGSSNLWVPSNRCSPWNEACRLHHRYDCEKSSTYKANGKPFSIQYGTGSVSG
                        :::::: :::::::::::::::::::::::::::::::::::::::::  ::::::::::::::::::::::::::::::::::::
AFRIPLTRFKSVRKQLAEEGIYIHEGPYPEPLVNLLDVVYYGPISIGTPPQDFQVIFDTGSANLWLPSSKCT--TKYCLHHHRYDSSKSSTYEADGRNFTIVYGSGNVEG

           *                                                   *                    ** *
VLSTDVVTVSSAKVQDQTFGEAINEPGLVFVVAKFDGILGLAFQSIAVDNVVPVFDNMISQGLVE-KPLFSVWLDRNDVQDIGGEIMFGGINKEHYMGDMYFVPLSSETY
::::::::::::::::::::::::::::::::::::::::::::::::::::::::::::::::: ::::::::::::.:::::::::::::::::::::::::::::::
FISKDVCRIGSAKVSGQPLGEALVVGGESLLEAPFDGILGLAYPSIAVDGVVPVFDNMMKQGLLGEQNVFSVYLNRDPSSKEGGEVLFGGIDHDHYKGSITYVPVTAKGY

WQIDLDGIQVT------SLTLCAQGCQAIVDTGTTLIVGPTADVNQLNEALGAVSIEGGLSVLECSQIYTLPPIEFSINGENLTLQPTDYVQEMSYRGGTICTSGFSGME
:::::::::::      :::::::::::::::::::::::::::::::::::::::::::::::::::::::::::::::::::::::::::::::.:::::::::::::
WQFHVDGVKSVSASKSAPELLCKDGCEAIANTGTSLITGPPEEVDSLNQYLGGTKTEGGQYLLDCDKLESLPNVTFTISGKEFSLRSKDYVLKVNQQGQTLCVSGFMGLE

TPGAPTWILGDVFIGAYYTVFDKEQRRVGFARST
::: ::::::::::::::::::::::::::::::
MPQ-PLWILGDVFLGPYYTIFDRDQDRVGFAEVA
```

```
CsCatD2's allosteric site: Q14, Y15, Y16, F32, A97, V149, E170, I171, F173
IrCatD's allosteric site: V39, Y40, Y41, F57, A120, L172, E193, V194, F196
```

**Figure S6**. Superposed results between CsCatD2$_{free}$ and IrCatD1 (PDB code: 5n7n). Based on the optimal superposition using TM-align [4], a potential allosteric site of CsCatD2$_{free}$ consists of Gln$_{14}$, Tyr$_{15}$, Tyr$_{16}$, Phe$_{32}$, Ala$_{97}$, Val$_{149}$, Glu$_{170}$, Ile$_{171}$ and Phe$_{173}$.

## References


1. Lovell, S.C.; Davis, I.W.; Arendall, W.B., 3rd; de Bakker, P.I.; Word, J.M.; Prisant, M.G.; Richardson, J.S.; Richardson, D.C. Structure validation by Calpha geometry: phi,psi and Cbeta deviation. *Proteins* **2003**, *50*, 437-450.
2. Wiederstein, M.; Sippl, M.J. ProSA-web: interactive web service for the recognition of errors in three-dimensional structures of proteins. *Nucleic Acids Res* **2007**, *35*, W407-410.
3. Colovos, C.; Yeates, T.O. Verification of protein structures: patterns of nonbonded atomic interactions. *Protein Sci* **1993**, *2*, 1511-1519.
4. Zhang, Y.; Skolnick, J. TM-align: a protein structure alignment algorithm based on the TM-score. *Nucleic Acids Res* **2005**, *33*, 2302-2309.
